# Redox state‐dependent modulation of plant SnRK1 kinase activity differs from AMPK regulation in animals

**DOI:** 10.1002/1873-3468.12852

**Published:** 2017-10-04

**Authors:** Bernhard Wurzinger, Andrea Mair, Katrin Fischer‐Schrader, Ella Nukarinen, Valentin Roustan, Wolfram Weckwerth, Markus Teige

**Affiliations:** ^1^ Department of Ecogenomics and Systems Biology University of Vienna Austria; ^2^ Department of Chemistry Faculty of Mathematics and Natural Sciences University of Cologne Germany; ^3^ Present address: Department of Biology University of Stanford Stanford CA USA

**Keywords:** AKIN10, protein kinase, redox regulation, reactive oxygen species, SnRK1

## Abstract

The evolutionarily highly conserved SNF1‐related protein kinase (SnRK1) protein kinase is a metabolic master regulator in plants, balancing the critical energy consumption between growth‐ and stress response‐related metabolic pathways. While the regulation of the mammalian [AMP‐activated protein kinase (AMPK)] and yeast (SNF1) orthologues of SnRK1 is well‐characterised, the regulation of SnRK1 kinase activity in plants is still an open question. Here we report that the activity and T‐loop phosphorylation of AKIN10, the kinase subunit of the SnRK1 complex, is regulated by the redox status. Although this regulation is dependent on a conserved cysteine residue, the underlying mechanism is different to the redox regulation of animal AMPK and has functional implications for the regulation of the kinase complex in plants under stress conditions.

## Abbreviations

**AMPK**, AMP‐activated protein kinase

***At***,* Arabidopsis thaliana*

**bZIP63**, basic region leucine zipper 63

**CBB**, coomassie brilliant blue

**CPK3**, calcium‐dependent protein kinase 3

**GSH**, reduced glutathione

**GST**, glutathione‐*S*‐transferase

**MAPK**, mitogen‐activated protein kinase

**MKK**, MAPK kinase

**NIA2**, nitrate reductase

**ROS**, reactive oxygen species

**SNF1**, sucrose nonfermenting kinase 1

**SnRK1**, SNF1‐related protein kinase

**vdwr**, van der Waals radius

In plants, the SNF1‐related protein kinase (SnRK1), an orthologue of the mammalian AMP‐activated protein kinase (AMPK) and yeast SNF1 protein kinase, plays a central role in regulating energy homeostasis, development and various stress responses 1, 2, 3, 4, 5, 6, 7. SnRK1 is evolutionarily highly conserved and orthologues can be found in all three domains of life 8. Like its mammalian and yeast counterparts, the SnRK1 protein kinase is a heterotrimeric complex consisting of one catalytic S/T protein kinase ‘alpha’ subunit, and the two regulatory ‘beta’ and ‘gamma’ subunits 9, 10. In *Arabidopsis thaliana* (*At*)*,* three genes encode for the SnRK1 alpha subunit isoforms. Two of them, AKIN10 (*At*SnRK1α1) and AKIN11 (*At*SnRK1α2) are ubiquitously expressed in vegetative tissue, while AKIN12 (*At*SnRK1α3) is predominantly expressed in pollen and seeds 11. Single knockout mutants of *akin10* and *akin11* produce viable offspring, whereas a respective double mutant is lethal 1, 4.

Much progress has been made in understanding how AMPK activity is regulated in mammals. There, the complex is under control of a multitude of factors, ranging from direct regulation via small effector molecules such as AMP/ADP/ATP over activity modulation by AMPK‐interacting proteins to more indirect regulation via hormone signalling 12. In plants, in contrast, the molecular mechanisms regulating SnRK1 activity are still hardly understood 13. Due to the obvious differences in physiology and metabolism between mammals, fungi and plants, many of the well‐studied regulatory mechanisms of AMPK and SNF1 seem not to be valid for plant SnRK1.

A common feature of the AMPK, SNF1 and SnRK1 catalytic subunits is their several hundred‐fold activation by phosphorylation of a threonine in the so‐called ‘activation loop’ (or T‐loop) which is conserved in all eukaryotic orthologues 4. This mechanism of activation is very similar to mitogen‐activated protein kinase (MAPK) activation by upstream MAPK kinases (MKKs) 14. In mammals, T‐loop phosphorylation of AMPK presents its major mode of activation and was shown to be highly dynamic and positively correlating with the AMP/ATP ratio‐dependent activity of the kinase 15. Binding of AMP to the gamma subunit of AMPK activates the complex by three mechanisms: (a) promotion of T‐loop phosphorylation by upstream kinases, (b) inhibition of T‐loop dephosphorylation by upstream phosphatases and (c) allosteric activation of the kinase 12. In plants, however, it is still unclear whether and how internal energy levels control the T‐loop phosphorylation status of SnRK1. Although AMP also inhibits dephosphorylation of the T‐loop in SnRK1, a phosphorylation‐promoting effect was not observed for the plant kinase complex [16]. Nevertheless, recent studies in vegetative leaves of Arabidopsis showed dynamics in SnRK1 T‐loop phosphorylation 1, 7. A drop of T‐loop phosphorylation was observed within the first 20 min of an extended night period which recovered immediately after 40 min of continued extended night treatment 1. A positive correlation between a rising AMP/ATP ratio and increasing T‐loop phosphorylation was so far only observed during the first hour of submergence of whole *At* plants 7. Surprisingly, a further increase of the AMP/ATP ratio after longer submergence was not reflected by enhanced T‐loop phosphorylation which, in fact, decreased again after the first hour of treatment 7. While these experiments demonstrate that the T‐loop phosphorylation status of plant SnRK1 is changing under certain conditions, they also indicate that something in addition to the AMP/ATP ratio influences SnRK1 kinase activity. Furthermore, data obtained from assaying the activity of whole SnRK1 complexes 17 suggest that SnRK1 is insensitive to allosteric activation by AMP. Importantly, the residues which were found to be critical for AMP‐dependent activation of AMPK are not conserved in SnRK1 9. Activation of SnRK1 via direct interaction with AMP, as observed for AMPK in animals, is therefore unlikely to happen, which leaves us with the question how the cellular status is relayed to SnRK1 in plants. In young tissues, this could in part be achieved by sugars, acting as small signalling molecules. Trehalose‐6‐phosphate (T6P), which is thought to act as a sugar‐deprivation signalling molecule, as well as glucose‐6‐phosphate (G6P) and glucose‐1‐phosphate (G1P) were found to have independent and potentially synergistic inhibitory effects on SnRK1 18, 19, 20. However, inhibition of SnRK1 activity by these sugar molecules requires an additional factor, which is only present in very young organs 19, 20. How energy availability could modulate T‐loop phosphorylation and SnRK1 activity in mature tissues is still an open question.

Here we report for the first time that AKIN10 activity is strongly dependent on the redox status *in vitro* and that this redox sensitivity is conferred by a single cysteine residue. Although the orthologous cysteine residue was found to be involved in mammalian AMPK redox regulation too 21, our data suggest that the molecular mechanism behind it differs considerably between plant AKIN10 and mammalian AMPK. Combining our results and published data on redox dynamics, we discuss the relevance of our findings on SnRK1 redox dependency in the cellular *in vivo* context for plants.

## Materials and methods

### Purification of recombinantly expressed proteins

*Arabidopsis thaliana* AKIN10 (splicing form 1/3), basic region leucine zipper 63 (bZIP63; splicing form 2), and calcium‐dependent protein kinase 3 (CPK3) were amplified from cDNA, subcloned into different expression plasmids by *Apa*I/*Not*I digestion and ligation, and transformed into the *Escherichia coli* strains ER2566 or BL21 for protein expression. Inactive versions of AKIN10 (K48M and T198A) and cysteine to serine mutants (C156S, C200S and C156/200S) were made by site‐directed mutagenesis. Primers for cloning and mutagenesis can be found in Table 1. The AKIN10 variants and bZIP63 (in pGEX‐4T; GE Healthcare, Little Chalfont, UK) as well as SnAK2 (in pDEST15; Thermo Fisher Scientific, generously provided by Elena Baena‐González 12) have an N‐terminal glutathione‐*S*‐transferase (GST) tag and were purified using Glutathione Sepharose as previously described in 2. CPK3 was cloned into a pTWIN expression vector with N‐terminal temperature/pH sensitive tag (NEB). Protein expression and purification were done as described in 22. C‐terminally truncated nitrate reductase (NIA2) was recombinantly expressed and purified as described in 23. For redox‐dependent kinase assays, elution buffers were exchanged for a basic kinase reaction buffer (50 mm Hepes, 2 mm MgCl_2_, pH 7.5) using PD MiniTrap G‐25 columns (GE Healthcare). Proteins were then concentrated using Vivaspin 500 Centrifugal Concentrators (GE Healthcare) and stored at −80 °C after adding glycerol to a final concentration of 10%.

**Table 1:** Primer table

	Forward primer	Reverse primer
CPK3 cDNA cloning	AAAAGGATCCGGGCCCATGGGCCACAGACACAGCAAGTCCAAATCCTCCG	TTTTGTCGACCTAGCGGCCGCACATTCTGCGTCGGTTTGGCACCAATTCTGGATTTCCC
AKIN10 T198A mutagenesis	GGTCATTTTTTGAAGGCTAGCTGTGGAAGTCC	AATGACCATCTCGCATTATGTTGCTCAGG
AKIN10 C156S mutagenesis	ATCAGGAGTGGAGTACTCCCATCGAAACATGG	CTCCTGATATTATCTGCTGAAAAAAGTTCC
AKIN10 C200S mutagenesis	GGTCATTTTTTGAAGACAAGTTCCGGAAGTCCAAATT	AATGACCATCTCGCATTATGTTGCTCAGG
AKIN10.1 cDNA cloning	GGGCCCATGGATGGATCAGGCACAGGCAGTA	GCGGCCGCAGAGGACTCGGAGCTGAGCAA
bZIP63.2 cDNA cloning	GGGCCCATGGAAAAAGTTTTCTCCGACGAAGAAATCTCC	TTGCGGCCGCCCTGATCCCCAACGCTTCGAATACG
AKIN10 K48M mutagenesis	GGTTGCTATCATGATCCTCAATCGTCG	GCAACCTTATGTCCTGTCAATGC

### *In vitro* kinase assays

Proteins were recombinantly expressed in *E. coli*. The ‘AIARA’‐peptide (AIARAASAAALARRR) was obtained from GenScript as chemically synthesised peptide. The final concentration for AIARA peptide in kinase assays was 100 μm. The kinase and substrate were incubated in kinase reaction buffer (50 mm Hepes, 2 mm MgCl_2_, 50 μm ATP, pH 7.5, plus 20 μm CaCl_2_ in assays with CPK3) supplemented with indicated concentrations of either DTT, reduced glutathione (GSH) or H_2_O_2_. For radioactive assays, 1 μCi P^32^ γATP were included in each reaction. Incubation times and temperatures were adjusted to *in vitro* kinase activity: assays with CPK3 were incubated for 10 min at room temperature, while assays with GST‐AKIN10 or GST‐SnAK2 were incubated for 30 min at 30 °C or room temperature (AIARA phosphorylation). For simulated oxidative burst assays, the respective kinases were mixed with their substrates in the presence of 3.5 mm GSH in kinase reaction buffer. Reactions were started by adding 1 μCi P^32^ γATP and the respective amount of H_2_O_2_ to reach the indicated H_2_O_2_ concentrations. For the sequential kinase assays presented in Fig. 4C, AKIN10 was first incubated in kinase reaction buffer containing 3.5 mm GSH and 50 μm ATP either with or without SnAK2 for 30 min to allow for saturating phosphorylation of AKIN10 by SnAK2. Subsequently, bZIP63 was added to the reactions together with 1 μCi P^32^ γATP, once maintaining 3.5 mm GSH and once adding 3.5 mm H_2_O_2_ to the reaction in the presence of 3.5 mm GSH. The secondary reactions were run for 15 min. Reactions were stopped by addition of 4× Laemmli buffer and boiling at 95 °C for 4 min. Proteins were then separated by SDS gel electrophoresis. Radioactive gels were dried and exposed on a Storage Phosphor Screen (GE Healthcare) before reading the signals with a Typhoon 8600 Variable Mode Imager (Amersham/GE Healthcare, Little Chalfont, UK). Nonradioactive gels were used for western blotting and detection of phosphorylation using phosphor‐specific antibodies: α‐P‐14‐3‐3: Phospho‐(Ser) 14‐3‐3 Binding Motif Antibody, #9601, Cell Signaling Technology (Danvers, MA, USA); and α‐P‐AMPK: Phospho‐AMPKα (Thr172) Antibody, #2531, Cell Signaling Technology. HRP‐conjugated secondary antibodies were used from GE Healthcare.

### Signal quantification with ImageJ

The ‘.gel’ files from the Typhoon Imager were imported in fiji (imagej; https://imagej.net/Fiji) 24 and regions of interest were defined to extract the signal intensity of each band. For GST‐bZIP63, the whole band was quantified and background‐subtracted. For assays with the ‘AIARA’ peptide, six equally sized circles were placed within each band (avoiding areas with strong background spots) and their intensities were summed up, followed by background subtraction and subtraction of the signal intensity from the control sample without kinase. Signal intensities of each assay were normalised to the 10 mm DTT sample before calculating the mean intensities of different experiments.

### Band shift assay for AKIN10 oligomerisation

The GST‐AKIN10 variants were incubated for 20 min at room temperature in kinase reaction buffer (50 mm Hepes, 2 mm MgCl_2_, pH 7.5) containing different concentrations of either DTT or H_2_O_2_ and either mixed with Laemmli buffer without or with 2‐mercaptoethanol. Samples containing 2‐mercaptoethanol were boiled for 5 min at 95 °C to improve breaking of disulphur bonds. The proteins were then separated by SDS gel electrophoresis, followed by western blotting with an antibody against AKIN10 (α‐AKIN10, AS10919; Agrisera, Umeå, Sweden).

### Protein sequence alignments

Protein sequences of all Arabidopsis proteins were taken from TAIR (www.arabidopsis.org). Sequences of other plant AKIN10 proteins were obtained from Phytozome (https://phytozome.jgi.doe.gov/pz/portal.html) by blasting the Arabidopsis AKIN10 protein sequence. In cases with more than one hit, only the one with the highest blast score or similarity to the Arabidopsis protein was considered. Sequences for human AMPKα1 and yeast SNF1α1 were downloaded from NCBI (https://www.ncbi.nlm.nih.gov/). Protein alignments were done in Geneious (Version 10) using the multiple alignment tool and selecting ClustalW alignment with default settings (Cost matrix: BLOSUM, Gap open cost: 10, Gap extend cost: 0.1). Box‐and‐line alignment schemes and short extracts of text alignment were exported as images.

### 3D models

For modelling the 3D structures of the *At*SnRK1 complex, the amino acid sequences of AKIN10 (AT3G01090.2), AKINβ1 (AT5G21170.2) and SNF4 (AT1G09020.1) were derived from TAIR 10 and subjected to modelling by SWISS‐MODEL heteroproject Beta server (https://swissmodel.expasy.org/interactive) 25. As templates for modelling, PDB (www.rcsb.org) 26 deposited structures 4rew and 4rer (heterotrimeric crystal structures) 27 as well as 4cfh 28 and 4qfg 29 (alpha subunit crystal structures) were used. Superposition of structures and images were created with the CCP4mg software (http://www.ccp4.ac.uk/MG/) 30.

### Gene identifiers

(a) *Arabidopsis thaliana* genes: AKIN10 (AT3G01090), bZIP63 (AT5G28770), CPK3 (AT4G23650), NIA2 (AT1G37130); (b) AKIN10 orthologues from human and yeast: AMPKα1 (gene bank: AAB32732.1), SNF1α1 (YDR477W, gene bank: KZV12718.1); (c) AKIN10 orthologues from selected plant species (Phytozome 11 identifiers): *Chlamydomonas reinhardtii* AKIN10 (Cre04.g211600.t1.1), *Physcomitrella patens* AKIN10 (Pp3c2_5790V3.5), *Selaginella moellendorffii* AKIN10 (80443), *Oryza sativa* AKIN10 (LOC_Os05 g45420.3).

## Results

In plants, redox changes of metabolites and proteins are an important integral part in signalling elicited by biotic and abiotic stimuli as well as in response to a changing energy balance 31, 32, 33. A recent publication linked AMPK kinase activity to Thrx1‐dependent redox regulation via conserved cysteine residues in its kinase domain and T‐loop 9, 21. It is therefore tempting to hypothesise that SnRK1, too, is connected to redox signalling processes.

**Figure 1:**
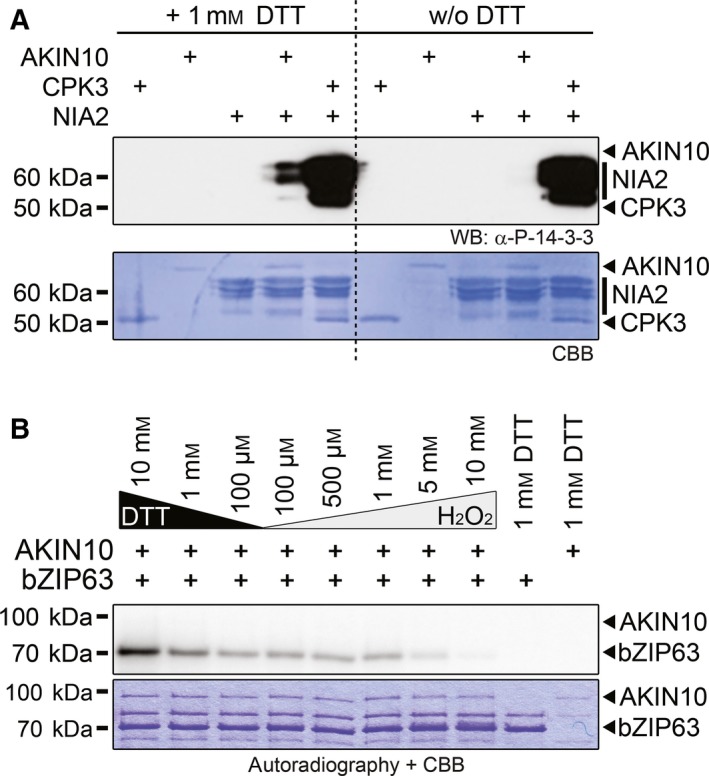
Arabidopsis AKIN10 but not CPK3 kinase activity is redox‐sensitive. (A) *In vitro* phosphorylation of the 14‐3‐3 protein‐binding site on NIA2 by AKIN10 and CPK3. Kinase reactions were done in the presence of 1 mm DTT or without DTT. Phosphorylation of NIA2 was visualised by western blotting with an antibody recognising a phosphorylated 14‐3‐3 protein‐binding motive only. The coomassie brilliant blue (CBB)‐stained membrane is shown below. Positions of the kinases and the substrate are indicated on the right‐hand side by black arrowheads and bars. (B) *In vitro* phosphorylation of bZIP63 by AKIN10. GST‐bZIP63 (bZIP63) was incubated with GST‐AKIN10 (AKIN10) in kinase buffer containing ^32^P γATP and different concentrations of either DTT or H_2_O_2_. The proteins were separated by SDS/PAGE and phosphorylated proteins were detected by subsequent autoradiography. The CBB‐stained gel is shown below. The positions of the full‐length proteins are indicated. Relative quantification of the signals is shown in Fig. S1B.

Indeed, we observed strong redox‐dependent changes in AKIN10 activity in *in vitro* phosphorylation assays with different substrates, supporting this idea. We first compared the phosphorylation of the previously described SnRK1 target *At*NIA2 34 in the presence and absence of 1 mm DTT. Remarkably, phosphorylation of the functionally important 14‐3‐3 binding site by AKIN10 required DTT (Fig. 1A). In contrast, the phosphorylation of the same residue by *At*CPK3 35, a member of the SnRK/CDPK group of plant protein kinases, was redox‐independent (Fig. 1A).

To further substantiate these findings, we performed a series of kinase assays covering a broad range of redox conditions, from strongly reducing (10 mm DTT) to strongly oxidising (10 mm H_2_O_2_) conditions, with *At*bZIP63, a recently well‐described *in vivo* substrate of AKIN10 2. Again, we observed higher AKIN10 activity under reducing conditions. The signal was strongest with 10 mM DTT, dropped to ~ 60% with 1 mm DTT and stayed stable at ~ 35% between 100 μm DTT and 1 mm H_2_O_2_, before almost vanishing at 5–10 mm H_2_O_2_ (Fig. 1B, Fig. S1B). Importantly, the same kinase assay series with CPK3, another known bZIP63 kinase 2, showed no redox dependency (Fig. S1A,B). As AKIN10 targets partly different residues on bZIP63 than CPK3 does, we wanted to exclude the possibility that the observed activity changes in the AKIN10 kinase assays are due to redox‐dependent conformational changes of the substrate. We therefore designed the AIARA peptide as novel artificial AKIN10 substrate in our assays. This peptide is similar to the well‐described AMARA 36 peptide substrate for AKIN10 but does not contain reducible/oxidisible amino acids under the tested conditions. These assays again confirmed the redox dependency of the AKIN10 kinase activity and redox insensitivity of the CPK3 kinase activity (Fig. S1C,D). It should be noted, though, that the AKIN10 redox state/kinase activity correlation blots look slightly different for assays with bZIP63 (Fig. S1B) and the AIARA peptide (Fig. S1D) – phosphorylation of the AIARA peptide decreased slower and more gradually than observed for bZIP63. This indicates that in case of bZIP63 also the substrate might undergo redox‐dependent conformational changes, which decrease its ability to get phosphorylated by AKIN10 but not by CPK3.

**Figure 2:**
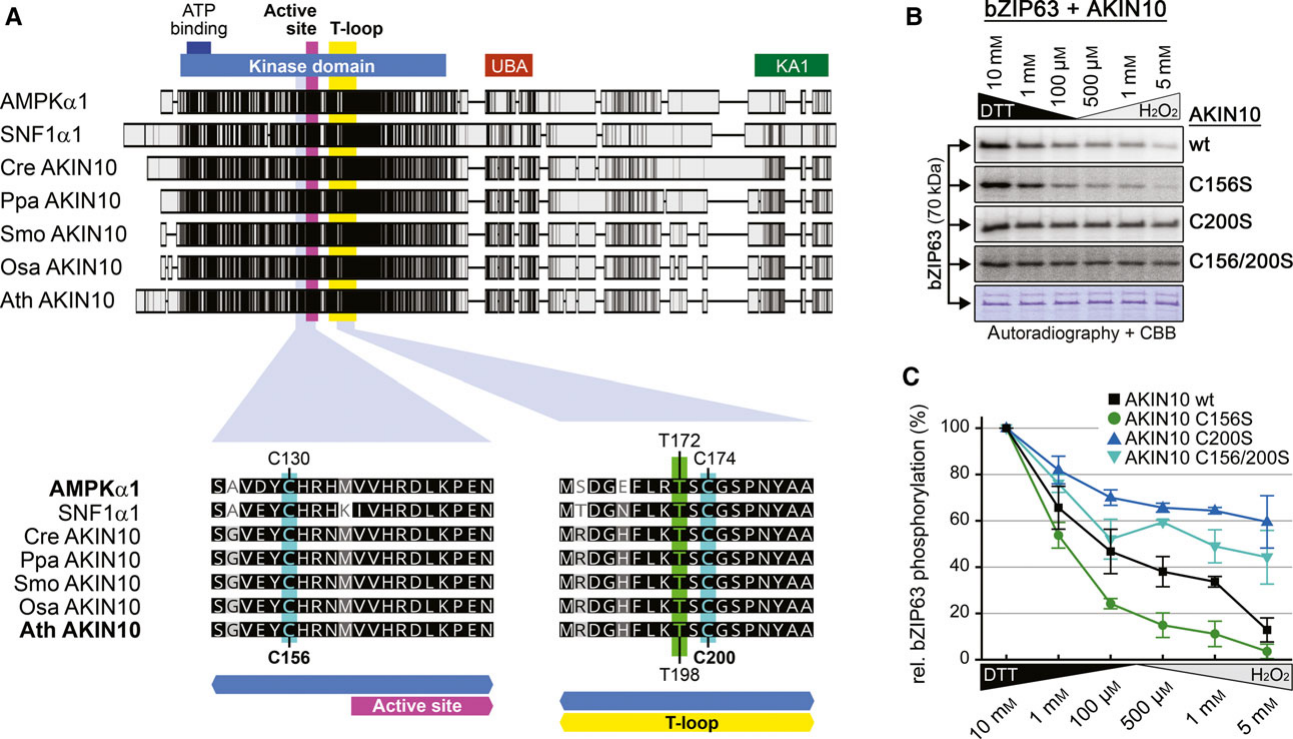
Redox sensitivity of AKIN10 partially depends on a conserved cysteine residue in its T‐loop. (A) Protein sequence alignment of AKIN10 orthologues in human (AMPKα1), yeast (SNF1α1) and plants. *Chlamydomonas reinhardtii* (Cre AKIN10), *Physcomitrella patens* (Ppa AKIN10), *Selaginella moellendorffii* (Smo AKIN10), *Oryza sativa* (Osa AKIN10) and *Arabidopsis thaliana* (Ath AKIN10) are shown as representatives of Chlorophytes, Bryophytes, Lycophytes, Monocots and Eudicots respectively. Sequences were aligned in Geneious using the default ClustalW settings. On the top, the complete sequence alignment is shown. The shaded boxes indicate the sequence conservation. The positions of the kinase domain (blue), the ATP‐binding site (dark blue), the active site (purple), the T‐loop (yellow), the ubiquitin‐associated domain (UBA, red) and the kinase‐associated 1 motif (KA1, green) are indicated. On the bottom, neighbouring sequences of C130 and C174 in AMPK are shown (highlighted in cyan). The crucial threonine in the T‐loop is highlighted in green. The shading indicates the degree of sequence similarity (black: 100%, dark grey: 80–100%, light grey: 60–80%, white: < 60%). An extended alignment of plant AKIN10 orthologues, including 52 different plant species, can be found in Fig. S2. (B) *In vitro* phosphorylation of bZIP63 by different AKIN10 variants. GST‐bZIP63 (bZIP63) was incubated with GST‐AKIN10 (wt) or three different cysteine to serine mutants (C156S, C200S and C156/200S) in kinase buffer containing ^32^P γATP and different concentrations of either DTT or H_2_O_2_. The proteins were separated by SDS/PAGE and phosphorylated proteins were detected by subsequent autoradiography. A representative coomassie brilliant blue‐stained gel is shown below. Relative quantification of three independent assays is shown in Fig. 2C. (C) Relative quantification of GST‐bZIP63 phosphorylation by GST‐AKIN10 (black squares) and its cysteine to serine variants (C156S, green circles; C200S, dark blue triangles; C156/200S, light blue triangles). The autoradiography values obtained from kinase reactions containing 10 mm DTT (highest kinase activity in all instances) were defined as 100%. The values are the mean ± standard deviation of three independent experiments.

We next wanted to identify the residues responsible for AKIN10 redox sensitivity. Shao *et al*. 21 reported that the residues C130 just before the active site and C174 in the T‐loop are the main sites involved in AMPK redox regulation. Both cysteines are evolutionarily conserved, also throughout the plant kingdom (Fig. 2A, Fig. S2), which made them the most interesting candidates for our analysis. The relative sequence position of the first cysteine is even retained in the closest homologues to the SnRK1 alpha kinases, the CIPK family, but not any more in the more distantly homologous cluster of the CPK family 37 (Fig. S3). In the AKIN10 splice variant 2, AMPK C130 and C174 correspond to AKIN10 C156 and C200 respectively. To investigate their roles for AKIN10 activity, we mutated either one or both cysteines to serine residues which are supposed to mimicking the reduced form of cysteine. The C156S mutant showed reduced kinase activity compared to the wild‐type (wt) AKIN10, which is not surprising given its close proximity to the active site. The redox sensitivity, however, was retained (Fig. 2B,C). On the contrary, the C200S mutant retained most of the wt activity but the redox sensitivity was abolished (Fig. 2B,C and Fig. S1C,D). The C156/200S double mutant combined both the overall reduction in activity as well as its insensitivity to the redox state (Fig. 2B,C). Therefore, we concluded that only C200 is required for the observed dynamic redox‐dependent changes in the kinase activity of AKIN10. This indicates a fundamental difference between the regulation of AKIN10 and AMPKα, as in AMPKα it was shown that the orthologous‐modified cysteines did not affect intrinsic kinase activity 21.

For AMPK, it was found that under oxidising conditions, C130 and C174 participate in intermolecular S‐S bond formation, leading to oligomerisation as well as to inhibition of AMPK activation by upstream kinases 21. For this reason, we looked at AKIN10 and found that it formed oligomers *in vitro* too (Fig. S4). In fact, only under strongly reducing conditions (10 mm DTT), a substantial amount of AKIN10 was present in its monomeric form. Under less reducing conditions, the majority of AKIN10 was present in high molecular weight complexes (Fig. S4). Surprisingly, neither C156 nor C200 seem to play a crucial role in AKIN10 oligomerisation, as the same behaviour was observed for the wt and all three C/S variants (Fig. S4). Combined with the fact that the C200S mutant is active even under oxidising conditions, when it is fully oligomerised, this suggests that the oxidation‐induced oligomerisation of AKIN10 is not responsible for its reduction in activity.

**Figure 3:**
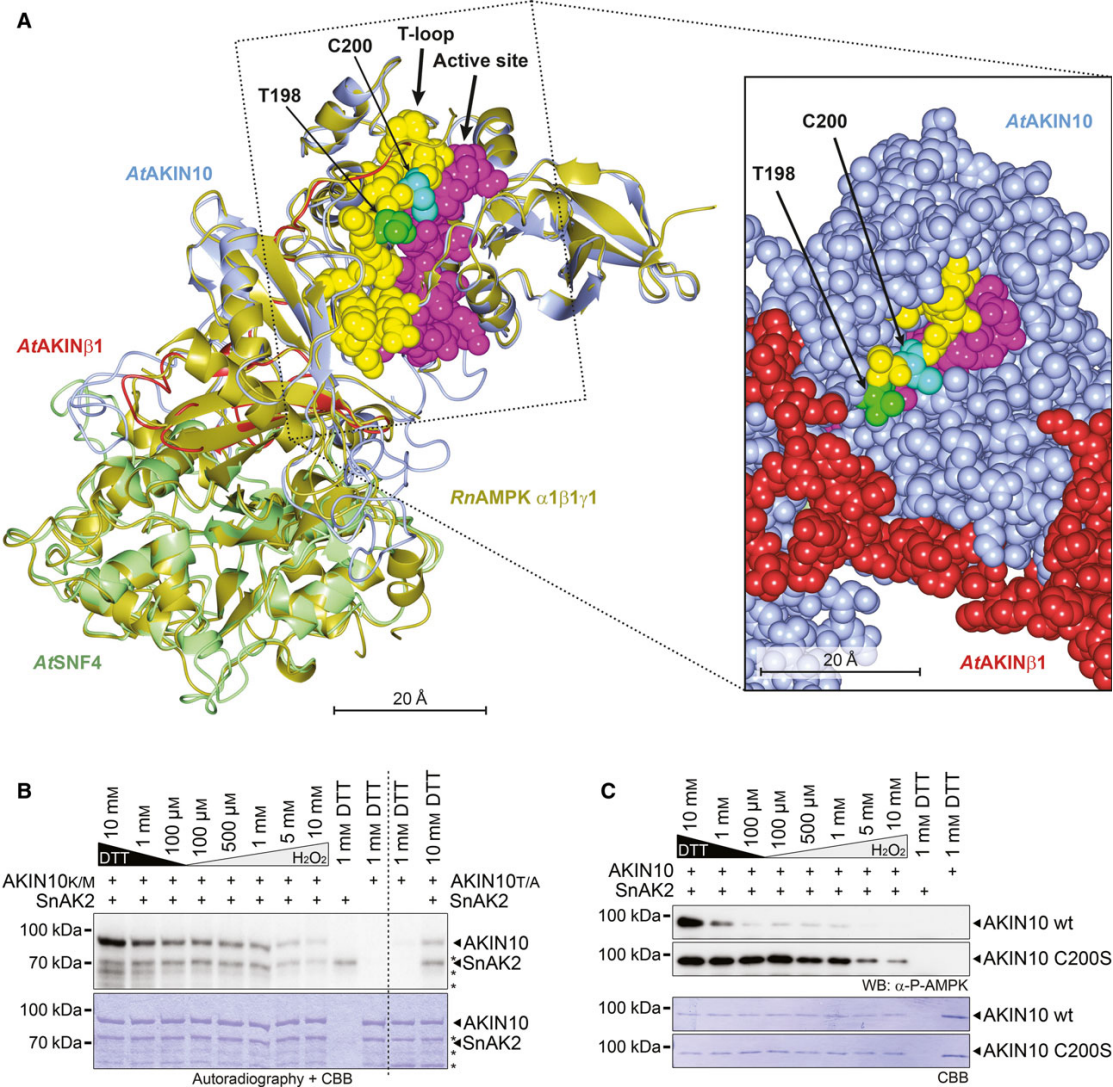
AKIN10 T‐loop phosphorylation by SnAK2 is redox‐dependent. (A) 3D‐structural model of the *At*SnRK1 complex based on the crystal structure of the rat *Rn* AMPK α1β1γ1 heterotrimeric complex. The left image shows a superposition of the *Rn* AMPK α1β1γ1 crystal structure and the derived *At*SnRK1 model. *Rn* AMPK α1β1γ1 is depicted in gold, *At* AKIN10 in blue, *At* AKINβ1 in red and *At* SNF4 in pale‐green. The T‐loop (yellow) with T198 (green) and C200 (cyan) and the active site (magenta) are highlighted. The image on the right‐hand side details the T‐loop and active site part of SnRK1 in a sphere‐representation with the spheres drawn to represent 1× van der Waals radius (vdwr). (B) *In vitro* phosphorylation of AKIN10 by SnAK2. Inactive versions of GST‐AKIN10 (K48M = AKIN10_K/M_ and T198A = AKIN10_T/A_) were incubated with its upstream kinase GST‐SnAK2 (SnAK2) in kinase buffer containing ^32^P γATP and different concentrations of either DTT or H_2_O_2_. The proteins were separated by SDS/PAGE and phosphorylated proteins were detected by subsequent autoradiography. The coomassie brilliant blue (CBB)‐stained gel is shown below. The positions of the full‐length proteins are indicated by arrowheads. Degradation products of GST‐AKIN10 are marked by asterisks. (C) *In vitro* phosphorylation of the AKIN10 T‐loop Threonine (T198) by SnAK2. Wild‐type GST‐AKIN10 (AKIN10 wt) or the C200S variant (AKIN10 C200S) was incubated with its upstream kinase GST‐SnAK2 (SnAK2) in kinase buffer containing different concentrations of either DTT or H_2_O_2_. Threonine 198 phosphorylation in the T‐loop of AKIN10 was visualised by western blotting with a phospho‐specific antibody (α‐P‐AMPK, top). The CBB‐stained membrane is shown below.

As C200 is in close vicinity to T198 in the T‐loop (Fig. 3A), the substrate for AKIN10‐activating kinase SnAK1 and 2 13, 38, 39, 40, 41, we asked if the C200 redox state would also influence AKIN10 phosphorylation by SnAK2. To this end, we first performed a series of kinase assays with SnAK2 and an inactive variant of AKIN10 (K48M) as substrate under different redox conditions. Using autoradiography for analysis, we observed that phosphorylation of AKIN10 by SnAK2 was highest under reducing conditions of 10 mm DTT in the reaction and gradually dropped towards more oxidising conditions (Fig. 3B), suggesting that reducing conditions could promote AKIN10 phosphorylation by its upstream kinases. Notably, under strongly oxidising conditions (5 and 10 mm H_2_O_2_), also SnAK2 autophosphorylation was reduced (Fig. 3B). This indicates that SnAK2 kinase activity, too, is redox‐dependent, albeit to a much lower extent than observed for AKIN10. The use of a T198A mutant as non‐activatable AKIN10 variant revealed that SnAK2 is also able to phosphorylate other residue(s) than T198 in AKIN10 (Fig. 3B). To obtain a more precise view on the T‐loop phosphorylation, we therefore analysed redox‐dependent AKIN10 phosphorylation by SnAK2 via western blotting, using an antibody which specifically recognises the phosphorylated T198. These assays basically confirmed the results from the autoradiography, demonstrating that T198 is most efficiently phosphorylated under highly reducing conditions (Fig. 3B). Interestingly, with the C200S variant as a substrate, SnAK2 could efficiently phosphorylate AKIN10 at T198 under all conditions (Fig. 3C). The moderate decrease of AKIN10 T198 phosphorylation at the two highest H_2_O_2_ concentrations might be attributed to the overall diminished SnAK2 kinase activity under these conditions (Fig. 3B,C). These findings are surprising as it was reported that AMPK activation by LkB1, the functional SnAK2 orthologue, was dependent on functional C174 and C130 side chains but was almost abolished when AMPK C174S/C130S variants were used in the kinase and interaction assays 21. This is remarkable as the T‐loop of AKIN10 belongs to the most conserved features of the AMPK/SNF1/SnRK1 kinase family on sequence level (Fig. 2A).

As DTT is no naturally occurring reductant in the plant cytosol, we wanted to make sure that our observations also apply for relevant in planta reductants such as GSH. We therefore tested the effect of GSH on AKIN10 activity and phosphorylation by SnAK2 in a series of kinase assays analogous to those shown in Figs 2B and 3B. Those assays confirmed that GSH, like DTT, is able to efficiently keep AKIN10 C200 in a reduced state, thereby resulting in full intrinsic AKIN10 kinase activity (Fig. S5A) as well as allowing the phosphorylation of T198 by SnAK2 (Fig. S5B).

**Figure 4:**
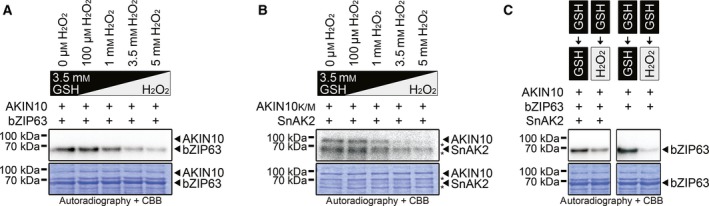
Intrinsic redox sensitivity of AKIN10 is retained in its T‐loop phosphorylated state. (A,B) *In vitro* kinase activity (A) and phosphorylation (B) of AKIN10 under a simulated H_2_O_2_ burst. GST‐AKIN10 (AKIN10 or inactive AKIN10_K/M_ = K48M) was first mixed with either its substrate GST‐bZIP63 (bZIP63) or its upstream kinase GST‐SnAK2 (SnAK2) in kinase buffer containing 3.5 mm GSH. The kinase reactions were then started by adding ^32^P γATP and rising concentrations of H_2_O_2_ as indicated. The proteins were separated by SDS/PAGE and phosphorylated proteins were detected via autoradiography. The coomassie brilliant blue (CBB)‐stained gel is depicted below. The positions of the full‐length proteins are indicated by arrowheads. Degradation products of GST‐AKIN10 are marked by asterisks. (C) Redox dependency of *in vitro* AKIN10 activity before and after phosphorylation by SnAK2. GST‐AKIN10 (AKIN10) was first prephosphorylated (left panel) or not (right panel) by SnAK2 in kinase buffer containing 3.5 mm GSH. Then, GST‐bZIP63 (bZIP63) and ^32^P γATP were added, either in the continued presence of 3.5 mm GSH (left lane) or in the presence of 3.5 mm GSH + 3.5 mm H_2_O_2_ (right lane). bZIP63 phosphorylation was analysed by autoradiography. The CBB‐stained gel is depicted below. As activated AKIN10 is ~ 25 times more active than nonactivated AKIN10, the autoradiographs were developed separately in order to avoid oversaturation of the image from activated AKIN10.

The notion that plant metabolism relies on a reducing cytoplasm 33, 42 suggests that under normal conditions, SnRK1 is present in its reduced form. This prompted us to ask if a simulated oxidative burst would lead to changes in AKIN10 activity. To this end, we set up a series of kinase assays in the presence of 3.5 mm GSH and added rising concentrations of H_2_O_2_. Indeed, with rising H_2_O_2_ concentrations, we observed an increasingly diminished intrinsic AKIN10 kinase activity (Fig. 4A), as well as a decrease in phosphorylation of AKIN10 by SnAK2 (Fig. 4B). This is similar to what we observed in the sequential redox series.

As the C200 redox state affects two distinct features of AKIN10 – the intrinsic AKIN10 kinase activity and its upstream kinase activation site – we asked if phosphorylation of T198 in the activation loop prior to an oxidative burst would protect AKIN10 from being inactivated by oxidation. Interestingly, this was not the case. Preincubation of AKIN10 with SnAK2 to fully phosphorylate T198 did not prevent a loss of AKIN10 kinase activity in response to H_2_O_2_ treatment (Fig. 4C). This suggests that oxidative bursts in the plant cell have the potential to modulate SnRK1 activity, regardless of its phosphorylation state, and could present a novel regulatory mechanism for SnRK1.

## Discussion

One major question following up this work is whether the described redox‐dependent activity changes of AKIN10 would be observable in the fully assembled *At*SnRK1 complex too. Structural modelling of the *At* SnRK1 subunits AKIN10, AKINβ1 and SNF4 on basis of the mammalian trimeric AMPK as well as AMPKα crystal structures shows that the C200 in the T‐loop resides on the surface of the protein and is unlikely to be blocked by domains of the regulatory beta or gamma subunits (Fig. 3A). Also, there is neither an SH group from AKIN10 nor from its beta or gamma subunits in the vicinity of C200, so that intramolecular C200S‐S‐R bond formation is highly unlikely to cause the observed effects. Comparison of structures obtained from modelling AtSnRK1 on the heterotrimeric AMPK complex and AMPK alpha subunits alone revealed that the AKIN10 core elements, such as the T‐loop and the active site of AKIN10, are highly similar in all analysed situations (Fig. S6). It was also shown that the regulatory beta and gamma subunits of the fully assembled SnRK1 complex do not interfere with AKIN10 activation by SnAK2 and that the kinase activities of the heterotrimeric SnRK1 complex and AKIN10 alone rise to similar levels when activated by SnAK2 43. Furthermore, AKIN10 targets used in this study were also identified as *in vivo* targets of the SnRK1 complex 1, 2, indicating that using the kinase subunit alone does not affect substrate recognition. Therefore, it is likely that our results obtained on the AKIN10 subunit are also valid for the heterotrimeric SnRK1 complex. Together, these data support our notion that the AKIN10 C200 oxidation state directly influences its kinase activity as well as the ability of SnAK2 to ‘recognise’ and efficiently phosphorylate the AKIN10 T‐loop also in the fully assembled SnRK1 complex.

The next question to be addressed is how the reported redox‐dependent AKIN10 activity dynamics would fit into the biological context. Plant metabolism generally relies on a reduced cytoplasm 33, 42. Under these conditions, we can assume that AKIN10 is present in a reduced form. However, local accumulation of reactive oxygen species (ROS) has been observed frequently in plants as a result of different environmental perturbations 44, 45. These ROS bursts indeed have the potential to oxidise cysteine SH‐side chains of different proteins 46, 47. In this respect, C200 oxidation of AKIN10, which as we have shown leads to its inactivation regardless of its phosphorylation state, may represent a part of the mechanism inactivating AKIN10, allowing the plant to terminate the AKIN10‐dependent stress response phase. A pending question connected to this proposed inactivation mechanism is if phosphatases dephosphorylating the AKIN10 T‐loop 48 would also be affected by the redox state of C200.

To underline the importance of our findings for AKIN10 regulation *in vivo*, it would be essential to show that critical AKIN10 cysteine side chains are dynamically reduced/oxidised also *in vivo*. In fact, first evidence that AKIN10 could be regulated by oxidation *in vivo* comes from cell culture experiments. In those, an oxidation‐specific interaction of AKIN10 with a yap1‐based probe for detection of sulfenylated cysteine side chains in proteins has been observed under oxidative stress (Frank van Breusegem, personal communication). However, it will be most important to identify which residues of AKIN10 exhibit dynamic oxidation states *in vivo*. Suitable methods to do so are being developed 46, 47, 49 and applying them specifically on AKIN10 will shed light on the extent of AKIN10 activity modulation at different cytosolic redox states.

In case that oxidation of C200 in AKIN10 takes place *in vivo* one may also wonder which oxidation state it will reach, either sulfenylation (−SOH), sulfinylation (−SO_2_H) or sulfonylation (−SO_3_H). −SO_3_H is regarded to be irreversible in biological systems, whereas −SO_2_H was reported to be reversible in some cases, aided by enzyme‐catalysed reactions 50. In this context, it would be interesting to know if in plants, specific interactions between AKIN10 and thioredoxins occur, which would lead to reduction of AKIN10, similarly as reported for AMPK regulation in mammals 21.

An interesting result of this study is that neither C156 nor C200 seem to be involved in redox‐dependent oligomerisation of AKIN10, which is contrary to what was observed for their orthologous residues C130 and C174 in mammalian AMPK 21. For AMPK, oligomerisation via C130‐C174 disulphide bond formation was even proposed as the mechanism for oxidation‐dependent inactivation 21. This we can exclude for AKIN10, which implies that the mechanism for oxidation‐dependent inactivation of AKIN10 must be a different one.

One puzzling fact about redox modulation of mammalian AMPK is that in one study, it was found to be deactivated under oxidising conditions 21, while in other studies, it was reported that oxidative stress leads to activation of AMPK via GST‐mediated S‐glutathionylation of the catalytic AMPK alpha and the regulatory AMPK beta subunits 51, 52. For AKIN10, it seems unlikely that S‐glutathionylation has a direct influence on C200‐mediated redox‐dependent activity changes. In our assays, the C200S mutant of AKIN10 shows a similar kinase activity under both reducing (DTT/GSH) and oxidising conditions (H_2_O_2_). This suggests that the reduced cysteine is sufficient for full AKIN10 activation. Furthermore, the decrease of AKIN10 activity by H_2_O_2_ in the oxidative burst assays (Fig. 4A) speaks against an activation of AKIN10 by spontaneous S‐glutathionylation of C200. A deactivation of AKIN10 by S‐glutathionylation of C200 is equally unlikely, as AKIN10 activity was reduced under oxidising conditions in assays not containing any glutathione (Fig. 2B). Still, at this point, we cannot exclude that SnRK1 activity may be regulated via GST‐catalysed S‐glutathionylation on other cysteines than C200 *in vivo*. Even a scenario like in mammals can be imagined where different redox‐mechanisms seem to modulate AMPK activity depending on the cell type the kinase is located in 21. However, to answer these questions, further experiments targeting eventually occurring S‐glutathionylation dynamics *in vivo* are necessary.

From the presented data, we can draw the conclusion that AKIN10 activity has the potential to be redox‐modulated *in vivo*. An estimation to what extent SnRK1 activity is redox controlled in the cell is currently difficult as the subcellular dynamics of H_2_O_2_ signalling are only starting to be understood 44. Along this line, a potential role of ‘redoxosomes’, a well‐established component in mammalian redox signalling 53, has only been superficially addressed in plants so far. On the other hand, GSH was demonstrated to exhibit more subcellular dynamics than previously assumed 54. Additionally, the subcellular localisation dynamics of SnRK1 itself adds to the complexity of this question. For yeast SNF1, transient complex formation at mitochondria has been described recently 55. Although it is accepted that the regulatory beta subunits are responsible for AKIN10 partitioning between nucleus and cytoplasm 10, detailed knowledge on the location of transient signalling complex formation, as has been observed in yeast, is still missing in plants. However, our data provide a solid basis for designing the further necessary experiments to elucidate the full extent of the described redox‐modulated AKIN10 activity in the plant itself.

## Author contributions

BW and MT conceived and supervised this study; BW, AM, KS and EN designed and performed the experiments; BW, MT and AM wrote the manuscript; BW, AM, KS, EN, VR, WW and MT revised the manuscript.

## Supporting information


**Fig. S1**. Arabidopsis AKIN10 but not CPK3 kinase activity is redox‐sensitive.

**Fig. S2**. Evolutionary conservation of AKIN10 C156, 200.

**Fig. S3**. Positional conservation of C200 and C156 in increasingly distantly related kinase families compared to SnRK1.

**Fig. S4**. AKIN10 redox‐dependent oligomerisation.

**Fig. S5**. AKIN10 C200 can be reduced by GSH *in vitro*.

**Fig. S6**. Core functional elements of AKIN10 are likely to be structurally similar in the SnRK1 heterotrimeric structure and the AKIN10 monomer.
