# Supplementary figures and images for: Redox state‐dependent modulation of plant SnRK1 kinase activity differs from AMPK regulation in animals
Source: FEBS Lett. 2017 Oct 4;591(21):3625–36. doi: 10.1002/1873-3468.12852 (PMC5698759; doi:10.1002/1873-3468.12852)

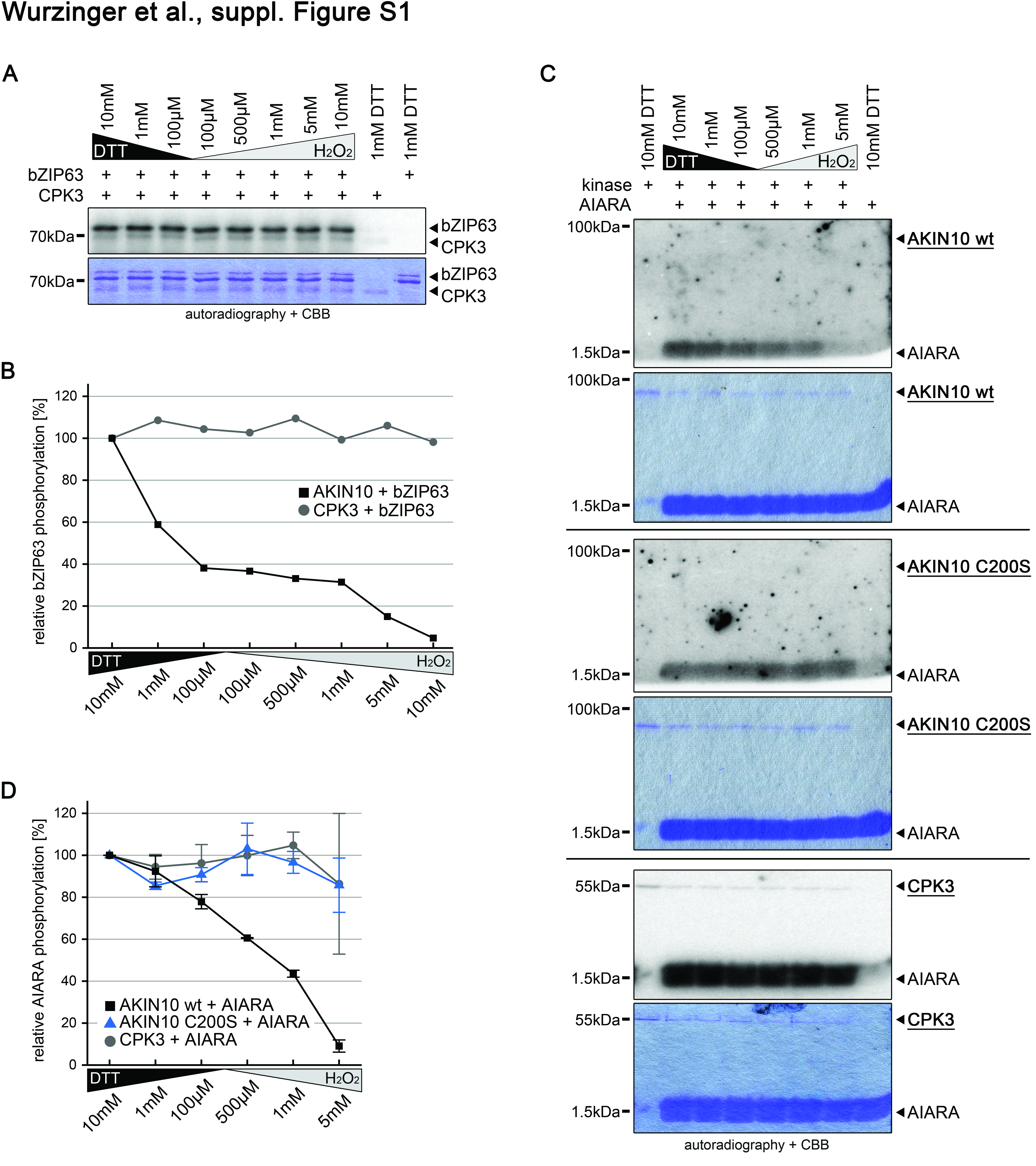

Supplement: Supplementary file 1 — Fig. S1. Arabidopsis AKIN10 but not CPK3 kinase activity is redox‐sensitive. [file FEB2-591-3625-s001.tif]

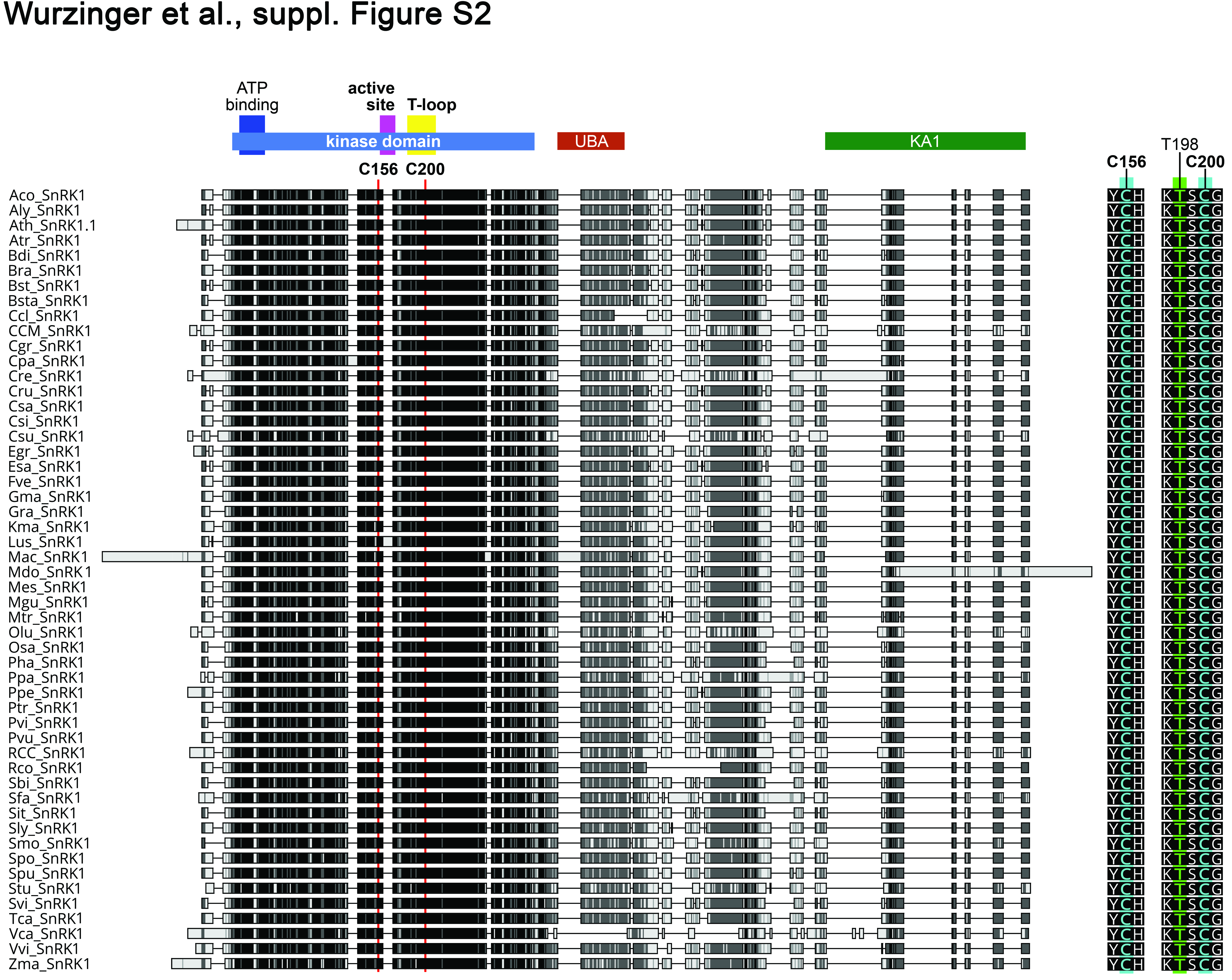

Supplement: Supplementary file 2 — Fig. S2. Evolutionary conservation of AKIN10 C156, 200. [file FEB2-591-3625-s002.tif]

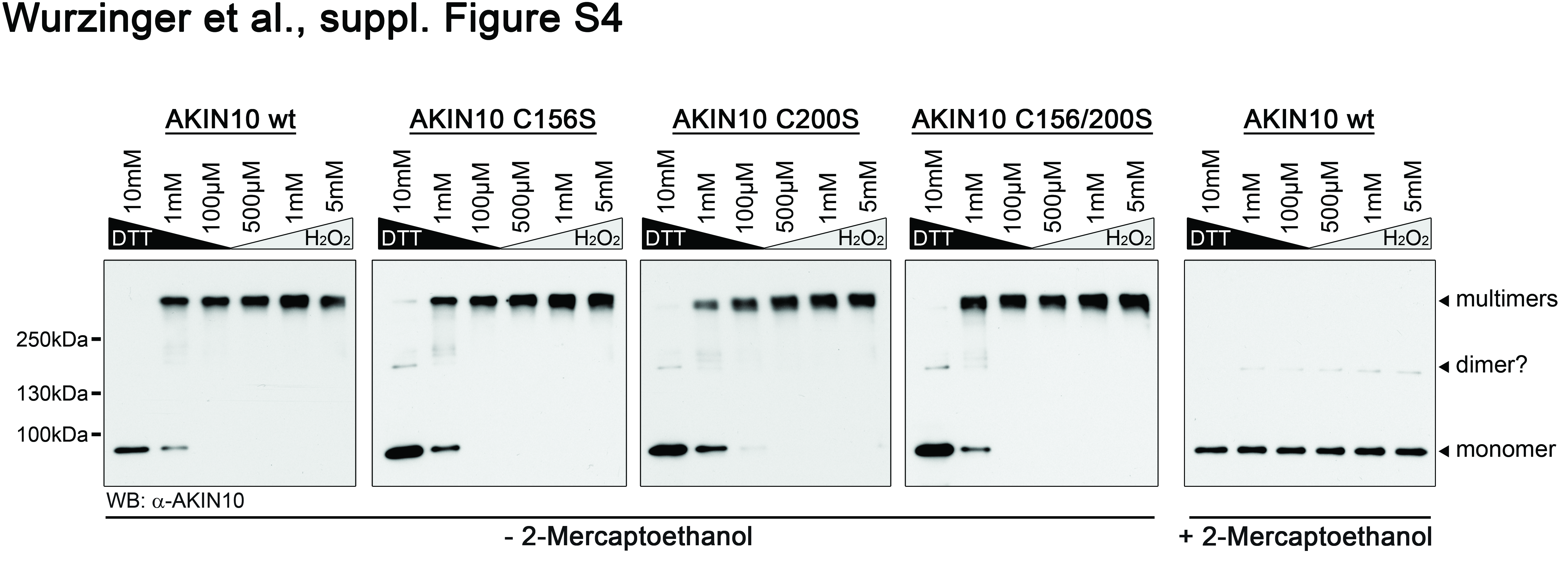

Supplement: Supplementary file 4 — Fig. S4. AKIN10 redox‐dependent oligomerisation. [file FEB2-591-3625-s004.tif]

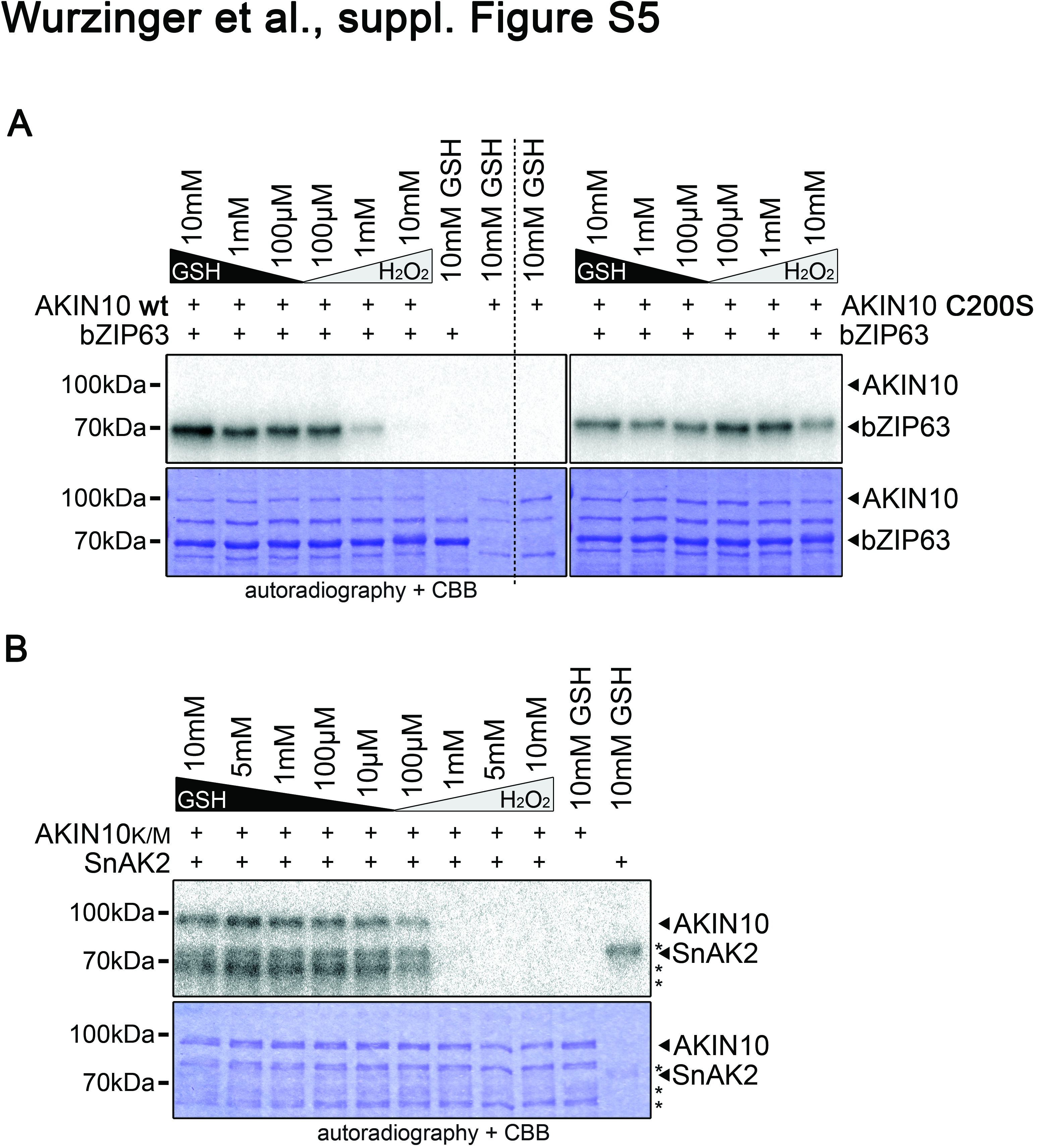

Supplement: Supplementary file 5 — Fig. S5. AKIN10 C200 can be reduced by GSH in vitro. [file FEB2-591-3625-s005.tif]

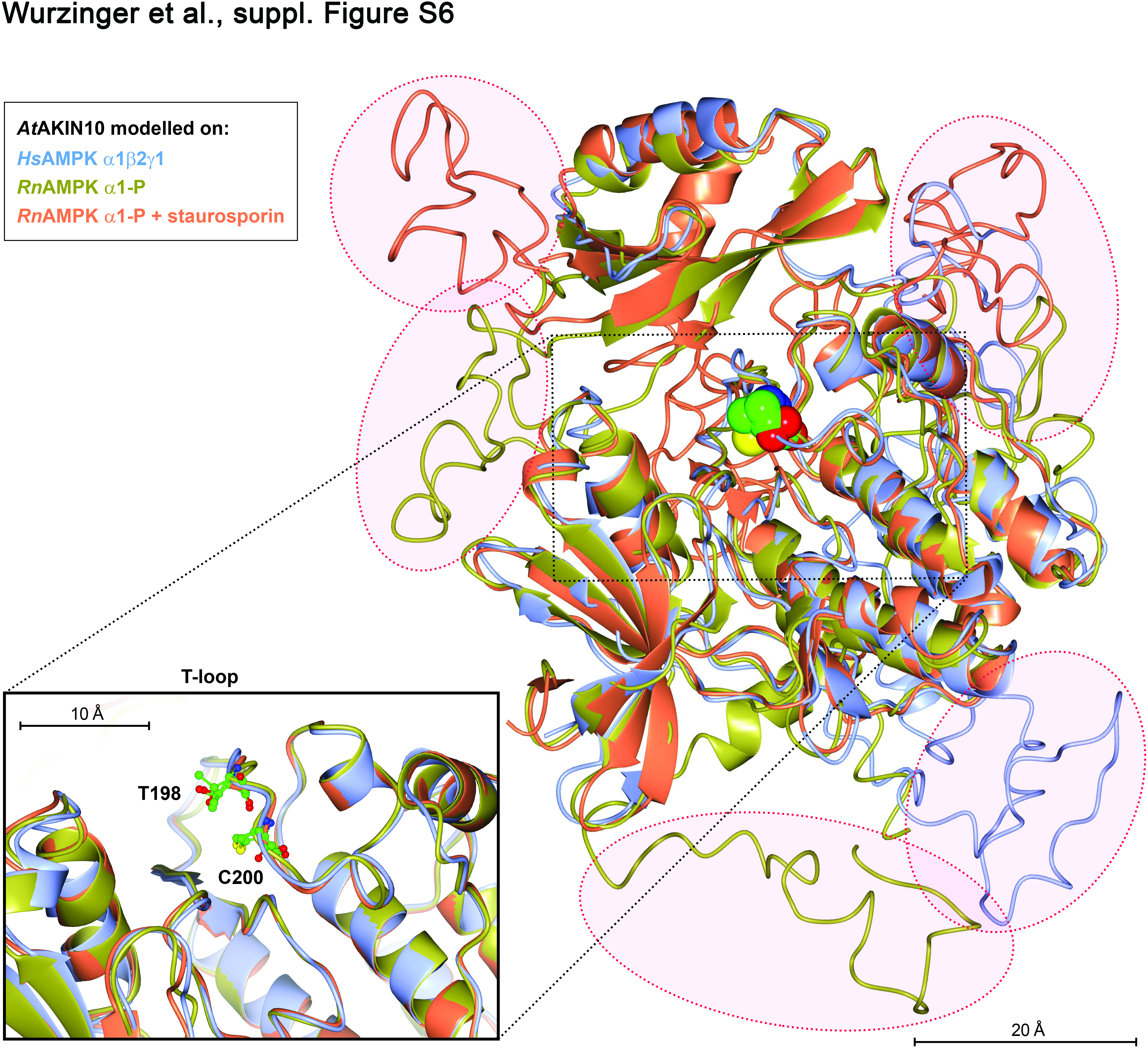

Supplement: Supplementary file 6 — Fig. S6. Core functional elements of AKIN10 are likely to be structurally similar in the SnRK1 heterotrimeric structure and the AKIN10 monomer. [file FEB2-591-3625-s006.tif]
